# Use of a symptom-based questionnaire to screen for the presence of significant voiding dysfunction in patients with multiple sclerosis and lower urinary tract symptoms: a pilot study

**DOI:** 10.1007/s00415-020-10068-2

**Published:** 2020-07-16

**Authors:** Vivien Li, Jalesh N. Panicker, Collette Haslam, Jeremy Chataway

**Affiliations:** 1 Queen Square Multiple Sclerosis Centre, Department of Neuroinflammation, UCL Queen Square Institute of Neurology, Faculty of Brain Sciences, University College London, London, UK; 2 Department of Uro-Neurology, The National Hospital for Neurology and Neurosurgery, Queen Square, London, UK; 3 Faculty of Brain Sciences, UCL Queen Square Institute of Neurology, University College London, London, UK; 4 National Institute for Health Research, Biomedical Research Centre, University College London Hospitals, London, UK

**Keywords:** Multiple sclerosis, Lower urinary tract symptoms, Management, Questionnaire

## Abstract

**Introduction:**

Lower urinary tract dysfunction is common in people with multiple sclerosis, leading to overactive bladder symptoms, voiding difficulties or a combination. First-line medications for overactive bladder symptoms are effective. Current guidelines recommend measuring post-void residual volume (PVR) before commencing these treatments, as they can potentially exacerbate voiding difficulties in those with significant underlying voiding dysfunction (pre-treatment PVR > 100 ml). However, facilities to do so are not readily available to all clinicians, potentially delaying effective therapy.

**Aims:**

To conduct a pilot study investigating the association between lower urinary tract symptoms and PVR volume in people with multiple sclerosis using a validated questionnaire and to determine if questionnaire scores can be used to exclude a significantly elevated (> 100 ml) PVR volume.

**Methods:**

Patients with multiple sclerosis referred to a tertiary hospital uro-neurology service completed the Urinary Symptom Profile questionnaire and underwent PVR measurement by bladder ultrasound. A ratio of the questionnaire low stream score/total score was calculated to standardise the relative degree of voiding symptoms compared to overall lower urinary tract symptoms.

**Results:**

Of 40 patients (29 females, mean age 50 years), 30% had an elevated PVR volume. PVR volume was correlated with low stream score and ratio of low stream/total score. A cut-off of > 0.15 for low stream/total score ratio had 92% sensitivity and 71% specificity in predicting an elevated PVR volume.

**Conclusion:**

A symptom-based questionnaire maybe a useful screening tool to distinguish patients in whom PVR measurement is required from those who could safely start on treatment for overactive bladder symptoms.

## Introduction

Lower urinary tract (LUT) dysfunction is common in multiple sclerosis (MS) affecting up to 75% of patients. Patients may experience different LUT symptoms depending on the site of demyelinating lesions in the central nervous system. Lesions of the suprapontine or spinal micturition pathways result in symptoms such as urinary urgency, urgency urinary incontinence, increased daytime frequency and nocturia, collectively known as storage or overactive bladder (OAB) symptom [1]. Urodynamic studies typically demonstrate detrusor overactivity. Dysfunction of the voiding phase can result from spinal cord lesions producing detrusor-sphincter dyssynergia where there is loss of coordinated activity between the detrusor and urethral sphincters. Less commonly, voiding dysfunction can arise due to detrusor underactivity. In both situations, symptoms include hesitancy, straining, slow and interrupted stream and incomplete bladder emptying [2].

Based on the UK consensus on the optimal management of LUT dysfunction in MS, all patients with MS presenting with new bladder symptoms should undergo measurement of the post-void residual (PVR) volume prior to starting oral agents for OAB symptoms, namely anti-muscarinic agents or β3-receptor agonist [3]. This is because subjective voiding symptoms are often unreliable [4]. Furthermore, the risks of missing an elevated PVR include increased risk for urinary tract infections and exacerbation of storage symptoms as the undetected voiding dysfunction could worsen following treatment commencement. However, facilities and equipment to measure PVR are not readily accessible to many neurologists, which may lead to delay in or never commencing highly effective treatment. One study surveying almost 10,000 patients with MS found 65% experienced at least one moderate LUT symptom, but only half received treatment with an anti-muscarinic medication [5].

A previous study by Milleman et al*.* found that clinical factors associated with an increased risk of elevated PVR in women with OAB symptoms include age older than 55 years, history of incontinence surgery, history of MS and stage 2 or greater vaginal prolapse [6]. However, there is a paucity of research into the association between LUT symptoms experienced by MS patients in particular and their PVR measurements. A simple tool that can be easily administered to patients with MS experiencing LUT symptoms to help distinguish those who are at high versus low likelihood of having an elevated PVR would be clinically useful.

## Aims

The aims of this pilot study are to investigate the association between LUT symptoms amongst patients with MS and their PVR measurements using a validated questionnaire and to determine if questionnaire scores could be used to exclude a significantly elevated (> 100 ml) PVR. This would potentially allow the questionnaire to be used to identify patients with an elevated PVR if bladder ultrasound was not available.

## Methods

The target population was patients aged 18 and over with a confirmed diagnosis of MS referred to the Uro-Neurology department at National Hospital for Neurology and Neurosurgery from April 2019 to January 2020. Patients were excluded if they were on medications to treat LUT symptoms, such as anti-muscarinic agents, β3-receptor agonists or α-receptor antagonists, required a urinary catheter (including intermittent self-catheterisation, urethral indwelling catheter or suprapubic catheter) and had other urological conditions, including urinary tract infection (based on urine dipstick performed on the sample produced for PVR measurement), or another neurological condition.

Data collected included patient demographics, MS subtype, duration of MS, use of disease-modifying therapies and duration of LUT symptoms. Patients completed the Urinary Symptom Profile (USP) (Box 1), a standardised validated 13-item questionnaire of LUT symptoms addressing three domains: stress urinary incontinence (SUI) (maximum score 9), overactive bladder (OAB) (maximum score 21) and low stream (LS) (maximum score 9) symptoms, with a greater score indicating worse symptoms [7]. To standardise the contribution of voiding symptoms represented by the LS score to the overall degree of LUT symptoms, the ratio of USP LS/total score was calculated.

Box 1 Urinary symptom profile [7] Stress urinary incontinence (SUI)

1. Over the past 4 weeks, please specify the number of times a week you had leaks during physical effort: (0 = no urine leaks, 1 = less than one urine leak a week, 2 = several urine leaks a week, 3 = several urine leaks a day)
  - During strenuous physical effort
  - During moderate physical effort
  - During light physical effort

Overactive bladder (OAB)

2. How many times a week have you had to rush to the toilet to urinate because you urgently needed to go? (0 = never, 1 = less than once a week, 2 = several times a week, 3 = several times a day)
3. When you have had an urgent need to urinate, for how many minutes on average have you been able to hold on? (0 = more than 15 min, 1 = from 6 to 15 min, 2 = from 1 to 5 min, 3 = less than 1 min)
4. How many times a week have you experienced a urine leak preceded by an urgent need to urinate that you were unable to control? (0 = never, 1 = less than once a week, 2 = several times a week, 3 = several times a day)
  - In the above case, what kind of leaks did you have? (0 = no leaks in this case, 1 = a few drops, 2 = light leaks, 3 = heavy leaks)

Over the last 4 weeks and under everyday conditions of social, professional or family life:

5. During the day, in general, how long elapsed between urinating? (0 = 2 h or more, 1 = between 1 and 2 h, 2 = between 30 min and 1 h, 3 = less than 30 min)
6. How many times on average have you woken up during the night by a need to urinate? (0 = never or once, 1 = twice, 2 = 3 or 4 times, 3 = more than 4 times)
7. How many times a week have you had a urine leak while asleep or have you woken up wet? (0 = never, 1 = less than once a week, 2 = several times a week, 3 = several times a day)

Low stream (LS)

8. How would you describe your usual urination over these past 4 weeks? (0 = normal, 1 = needed to push with abdominal (stomach) muscles or lean forward (or required a change of position) to urinate, 2 = needed to press on the lower stomach with my hands, 3 = used a catheter)
9. In general, how would you describe your urine flow? (0 = normal, 1 = weak, 2 = drop by drop, 3 = used a catheter)
10. In general, how has your urination been? (0 = normal and quick, 1 = difficult to start then normal, 1 = easy at first but slow to finish, 2 = very slow from start to finish, 3 = used a catheter)

All patients underwent non-invasive uroflowmetry with determination of maximum urinary flow rate (Qmax) and measurement of PVR in clinic via ultrasound by trained nursing staff (Albyn Medical Smartflow, Bardscan Portable Ultrasound). PVR was classified as elevated if it exceeded 100 ml [3].

### Statistical analysis

Statistical analysis was performed in IBM SPSS Statistics version 25.* T* test was used to compare means between groups. Pearson test was used to assess correlation between variables (PVR and questionnaire scores, PVR and Qmax). Linear regression was used to analyse the relationship between independent variables to the dependent variable of PVR. Fisher’s exact test was used to analyse the relationship between categorical variables, including PVR [classified as high (> 100 ml) or normal (< 100 ml)]. A level of p < 0.05 was considered statistically significant.

Assessments were conducted as part of routine clinical management. Patients provided written informed consent for anonymised questionnaire data to be used.

## Results

### Demographics

A total of 40 patients (29 female) completed both the USP questionnaire and PVR measurement. Of these 30 (75%) were Caucasian. Mean (SD) and median (IQR) age were 50 (13) years and (41–60) respectively, with no significant difference between females (mean 51, SD 12 years) and males (mean 50, SD 16 years). Mean (SD) and median (IQR) duration since MS diagnosis was 18 (13) and 14.5 years (9–24) respectively. 28 (70%) had relapsing remitting MS, 7 (17.5%) had secondary progressive MS and 5 (12.5%) had primary progressive MS. 17 (43%) were currently on disease-modifying therapy. Mean (SD) and median (IQR) duration of LUT symptoms was 7.8 (5.9) and 6.5 (3–12) years respectively. Mean age of patients with RRMS was significantly lower than those with progressive MS [47 vs. 61 years, *p* = 0.002, 95% confidence interval (CI) 5.2–22]. However, there was no statistically significant difference in duration since MS diagnosis (15 vs. 23 years) nor duration of LUT symptoms (7.8 vs. 8.0 years). There were no significant sex differences in proportion with relapsing remitting versus progressive MS and duration of MS and LUT symptoms.

### USP score and PVR measurement

Mean USP questionnaire SUI, OAB, LS and total scores were 0.78 (range 0–7), 6.4 (range 0–18) and 1.6 (range 0–4), 8.8 (range 0–22) respectively, indicating a mix of storage and voiding LUT symptoms.

Mean (SD, range) PVR was 88 (103, 0–387) ml and was greater than 100 ml in 12 patients (30%). There was a significant difference in mean PVR between the group of patients with PVR of below or above 100 ml (33 ml versus 218 ml, mean difference 185 ml, 95% confidence interval [CI] 145-225 ml, p < 0.001). Mean (SD) Qmax was 20 (16) ml/s and there was no significant correlation between PVR and Qmax (r^2^ = 0.12, *p* = 0.08). Characteristics of patients with PVR below or over 100 ml is shown in Table 1. Only mean USP LS score and USP LS/total score demonstrated significant differences between the two groups. There was trend towards a significant difference in sex distribution (*p* = 0.056).

**Table 1:** Characteristics of patients (n = 40) with PVR below or over 100 ml

	PVR < 100 ml	PVR > 100 ml	*p* value, mean difference, 95% CI
Sex [females (%)]	23 (58%)	6 (15%)	
Age (mean, SD; median, IQR)	52 (11), 55 (43–60)	50 (17), 47 (34–62)	
MS subtype [RRMS (%), SPMS (%), PPMS (%)]	20 (50%) 4 (10%) 4 (10%)	8 (20%) 3 (7.5%) 1 (2.5%)	
MS duration, years (mean, SD; median, IQR)	18 (12), 16 (9–25)	17 (14),12 (7–16)	
LUT symptom duration, years (mean, SD; median, IQR)	7.3 (6.4), 5 (2.8–12)	8.9 (4.9), 10 (4–11)	
USP SUI score (mean, SD; median, IQR)	0.79 (1.5), 0 (0–1)	0.75 (1.2), 0 (0–1.3)	
USP OAB score (mean, SD; median, IQR)	6.4 (3.5), 6 (4–8)	6.4 (4.1), 6 (3.3–9)	
USP LS score (mean, SD; median, IQR)	1.1 (1.2), 1 (0–2)	2.8 (1.2), 3 (2–4)	*p* < 0.001, 1.7, 0.88–2.6
USP total score (mean, SD; median, IQR)	8.3 (4.6), 8 (5–10)	10 (5.4), 9 (7–11)	
USP LS/total score (mean, SD; median, IQR)	0.15 (0.20), 0 (0–0.23)	0.32 (0.21), 0 (0.17–0.39)	*p* = 0.019, 0.17, 0.029–0.31

*PVR* post-void residual volume, *CI* confidence interval, *SD* standard deviation, *RRMS* relapsing–remitting multiple sclerosis, *SPMS* secondary progressive multiple sclerosis, *PPMS* primary progressive multiple sclerosis, *LUT* lower urinary tract, *USP* urinary symptom profile, *SUI* stress urinary incontinence, *OAB* overactive bladder, *LS* low stream

### Predictors of PVR

**Fig. 1:**
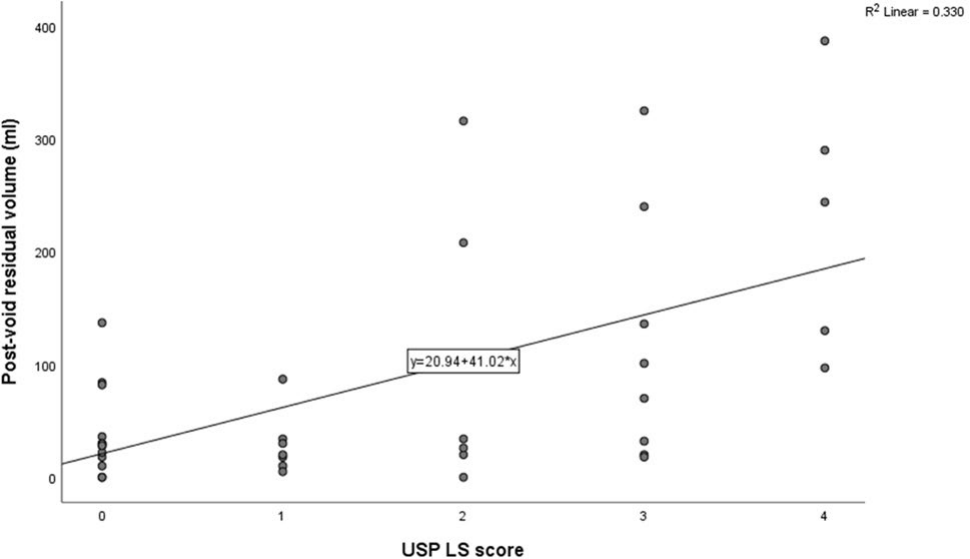
Correlation between USP low stream score and post-void residual volume

Based on univariate analyses, patient age, duration of MS, MS subtype and duration of LUT symptoms were not associated with PVR. PVR was correlated with USP LS score (*r* = 0.573, *p* < 0.001) (Fig. 1) and USP LS/total score (*r* = 0.422, *p* = 0.007), but not SUI or OAB scores. Males had significantly higher PVR (mean 165 ml vs. 60 ml, mean difference 105 ml, 95% CI 39–171, *p* = 0.003) and USP LS score (mean 2.36 vs. 1.34, mean difference 1.02, 95% CI 0.03–2.01, *p* = 0.045) than females.

**Fig. 2:**
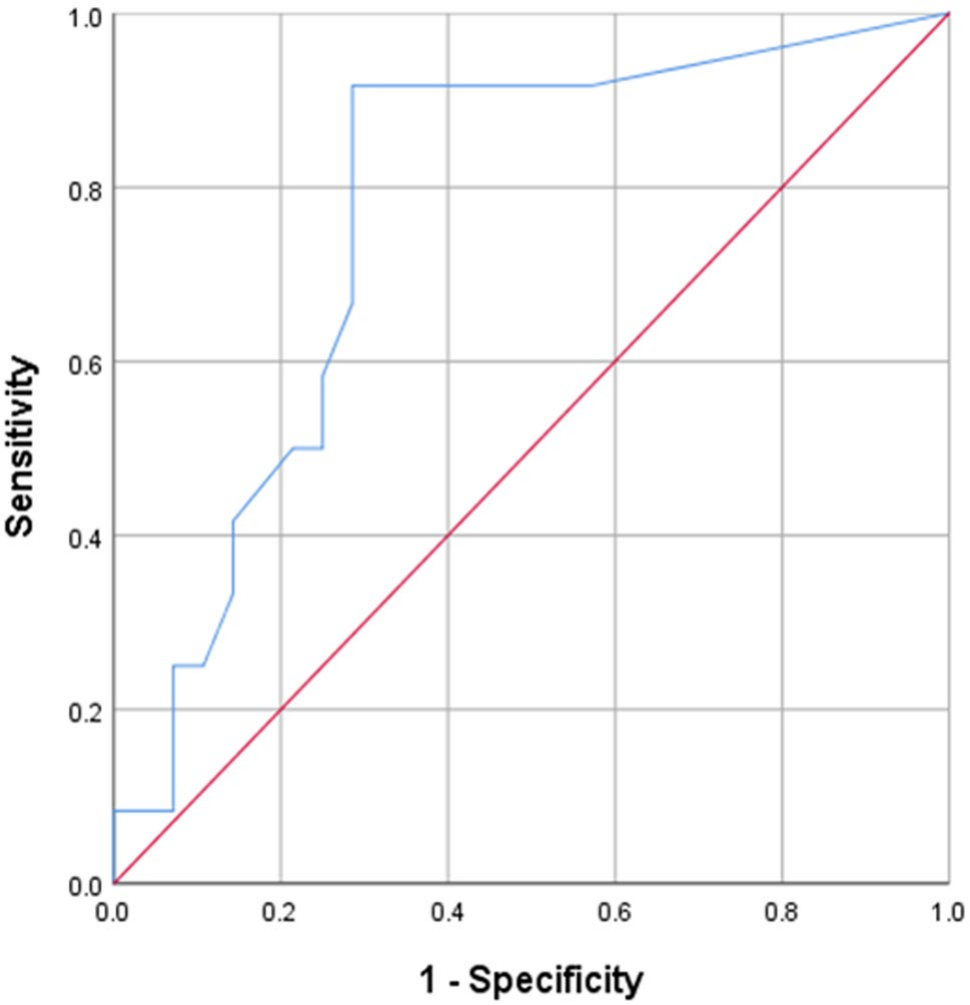
Receiver operating characteristics curve for USP low stream (LS)/total score ratio in predicting a post-void residual volume of over 100 ml

In order to determine the ability of the USP questionnaire, specifically the USP LS/total score ratio, to predict a PVR of over 100 ml, area under the curve (AUC) of the receiver operating characteristics (ROC) curve was calculated (Fig. 2). AUC was 0.77 (95% confidence interval 0.62–0.93, *p* = 0.007). A cut-off value of 0.15 produced a sensitivity of 92% and false positive rate of 29%. Amongst this cohort, only one patient with a PVR of over 100 ml had a ratio below 0.15 and was misclassified (i.e., false negative).

## Discussion

Our study found that using a USP LS/total score ratio of 0.15 as a cut-off has good sensitivity in predicting patients with MS who have a PVR exceeding 100 ml. For healthcare professionals encountering the large number of patients with MS who have OAB symptoms and are considering an anti-muscarinic agent or β3-receptor agonist, but do not have direct or immediate access to facilities to measure the PVR, this short symptom-based patient questionnaire can provide a way to distinguish between patients in whom treatment can be initiated without delay from those who require PVR measurement first. Whilst the false positive rate of 29% is relatively high and these patients may be subjected to a degree of inconvenience as a result of waiting for a formal PVR measurement to be carried out, this is a non-invasive and essentially risk-free investigation.

Traditionally it has been thought that whilst storage LUT symptoms are generally a good guide as to the presence of detrusor overactivity, they are less reliable when there is additional voiding dysfunction and incomplete emptying [4]. Interestingly our study found that USP LS score was moderately correlated with PVR volume. Previous studies assessing the relationship between neurological and urological symptoms as well as subjective urinary symptoms versus objective urological measurements have produced mixed findings. One study which assessed EDSS and functional system scores (FSS), clinical LUT symptoms and filling cystometry in patients with MS found that the degree of lower limb pyramidal weakness correlated with severity of urological symptoms in general, as both are thought to reflect the extent of spinal involvement. However, only 47% of patients with a significantly raised PVR reported a sensation of incomplete bladder emptying, suggesting that lack of voiding LUT symptoms cannot sufficiently exclude the presence of a high PVR accurately [8].

In another study, an EDSS pyramidal or bladder/bowel FSS of ≤ 1 was found to be not associated with incomplete bladder emptying, suggesting that it may not be necessary to measure the PVR in such patients [9]. However, the opposite findings have also been reported in another larger study which found no association between EDSS bladder/bowel FSS and measured PVR [10]. One weakness of using the EDSS is low intra-rater and inter-rater reproducibility, especially for patients with mild to moderate disability [11]. Furthermore, as the bladder/bowel FSS only ranges from 0–3, it does not sufficiently delineate the severity nor fully encapsulate the nature of symptoms. Severity of LUT symptoms as assessed by unstructured interview also did not predict objective measures of LUT dysfunction including PVR, Qmax and the presence of detrusor-sphincter dyssynergia [12].

Whilst numerous questionnaires on bladder symptoms are available, the USP was chosen as it is a validated questionnaire that incorporates different domains of LUT symptoms and better describes the frequently mixed symptoms experienced by patients with MS. Although not specifically validated in neurological patients, it has been recommended as a screening tool for LUT symptoms in MS [13] and used as an outcome measure in previous studies of patients with MS [14–16] and other neurological conditions [17, 18]. A ratio of the USP LS/total scores was calculated to account for the degree of voiding LUT symptoms compared to a patient’s overall profile of LUT symptoms.

The limitations of our study are the relatively small number of individuals who underwent assessment, but the demographic characteristics of our patient population was within the range of the previously reported studies [8–10, 12]. Patients were also taken from those referred to a tertiary uro-neurology service, so may have more severe LUT symptoms than the overall population of patients with MS. However, as referrals are generally made for those with significant symptoms in whom more advanced investigation and treatment is being considered pending evaluation such as PVR measurement, these may also be the individuals in whom such a tool is most valuable. Our study only used one PVR measurement and this can fluctuate over time and on different occasions. Therefore, repeated measurements would be useful to further validate this finding. Invasive urodynamics data was only available in a small proportion of our cohort, so we were not able draw conclusions on the relationship between these findings, questionnaire responses and PVR. In the context of MS, the aetiology of an elevated USP LS score was assumed to be neurogenic LUT dysfunction such as detrusor-sphincter dyssynergia or detrusor underactivity. However, given the mean age of this population of 51 years, other non-neurological causes such as benign prostatic hypertrophy may also contribute to a raised PVR, which was found to be higher in the male patients.

As this is a pilot study, further validation in a larger cohort, including patients of different ages and levels of disability, and different settings, such as in primary care or general neurology and MS clinics, would be required before this tool could be used in practice. All patients commenced on an anti-muscarinic medication or β3-receptor agonist for OAB symptoms do still require regular monitoring and prompt review is advised if patients experience symptoms suggestive of a deterioration in voiding function, such as a paradoxical worsening of OAB symptoms or recurrent urinary tract infections. While a ratio of the USP LS/total score has not been used in previous studies, the pilot data from our study raise the possibility of using a simple validated questionnaire as a screening tool to distinguish patients in whom further urological investigations including PVR measurement is required from those that could be safely started on treatment for OAB symptoms. This could reduce the delay in initiating first-line therapy for these frequently disabling but treatable symptoms.

## Data Availability

Anonymised data will be shared by request from qualified investigators.
